# The caudal pedunculopontine tegmental nucleus may be involved in the regulation of skeletal muscle activity by melanocortin-sympathetic pathway: a virally mediated trans-synaptic tracing study in spinally transected transgenic mice

**DOI:** 10.18632/oncotarget.17983

**Published:** 2017-05-18

**Authors:** Zhi-Gang He, Bao-Wen Liu, Zhi-Xiao Li, Xue-bi Tian, San-Guang Liu, Anne Manyande, Ding-Yu Zhang, Hong-Bing Xiang

**Affiliations:** ^1^ Department of Anesthesiology and Pain Medicine, Tongji Hospital of Tongji Medical College, Huazhong University of Science and Technology, Wuhan, Hubei, People's Republic of China; ^2^ Department of Hepatobiliary Surgery, The Second Hospital, Hebei Medical University, Shijiazhuang, People's Republic of China; ^3^ School of Human and Social Sciences, University of West London, London, UK; ^4^ Intensive Care Unit, Wuhan Medical Treatment Center, Wuhan, P. R. China

**Keywords:** pedunculopontine tegmental nucleus, melanocortin-4 receptor, skeletal muscle, pseudorabies virus, mouse

## Abstract

Understanding neuroanatomical sympathetic circuitry and neuronal connections from the caudal pedunculopontine tegmental nucleus to skeletal muscle is important to the study of possible mechanisms of pedunculopontine tegmental nucleus (PPTg) and cuneiform nucleus (CnF) that are involved in the regulation of skeletal muscle activity of the sympathetic pathway. The aim of this study was to use virus PRV-614 to trace the melanocortin-sympathetic neural pathways from PPTg and CnF to a hindlimb muscle (gastrocnemius) in spinally transected MC4R-GFP transgenic mice. PRV-614 was injected into the gastrocnemius muscle after receiving a complete spinal cord transection below the L2 level. PRV-614/MC4R-GFP and PRV-614/TPH dual-labeled neurons were found in the dissipated parts of PPTg (dpPPTg), but not between the compact parts of PPTg (cpPPTg) and CnF. It is proposed that a hierarchical pathway of neurons within the caudal pedunculopontine tegmental nucleus sends projections to the RVLM, which in turn projects onto the IML sympathetic preganglionic neurons that regulate muscle blood flow through melanocortin-sympathetic signals. Our results collectively indicate that MC4Rs expressed in caudal pedunculopontine tegmental nucleus may be involved in skeletal muscle activity of melanocortin-sympathetic pathways.

## INTRODUCTION

Much evidence supports the claim that cholinergic neuronal loss within the mesencephalic pedunculopontine tegmental nucleus (PPTg) is associated with the level of dopaminergic degeneration [1] and the clinical severity of the characteristic motor symptoms of Parkinson's disease: postural instability and gait disturbances [2, 3]. PPTg deep brain stimulation (DBS) used to treat these symptoms has also provided some insight into the function of PPTg [4–6]. Studies that evaluated the safety and efficacy of PPTg DBS, failed to establish the optimal site and precise effects of PPTg DBS of certain motor symptoms of Parkinson's disease. In an earlier study, we reported the existence of a direct neuronal circuit from the caudal pedunculopontine tegmental nucleus and cuneiform nucleus (CnF) to the skeletal muscle through the motor pathway [7]. It is however, unclear whether these effects of PPTg DBS are partially mediated through the sympathetic connections of this neuronal circuit. Clearly, it is important to study the possible mechanism of pedunculopontine tegmental nucleus and cuneiform nucleus involved in the regulation of skeletal muscle activity of the sympathetic pathway.

There are reports that the pedunculopontine tegmental nucleus which coexpresses choline acetyltransferase (a marker of cholinergic neurons) immunoreactivity and melanocortin-4 receptor (MC4R) expression [8, 9], is involved in mechanisms of behavioral state control, locomotion and muscle tone control [7, 10, 11]. In addition, the central melanocortin system has also been found to be a critical mediator of skeletal muscle activity [12]. Other studies have shown that central regulation of sympathetic nerve activity is a major component of melanocortinergic action [8, 13, 14] and that central serotonergic and catecholaminergic positive neurons regulate sympathetic outflow [15, 16]. More recently, MC4Rs expressed by cholinergic neurons (including sympathetic preganglionic motor neurons) [17] in the central nervous system were shown to be key regulators of energy and glucose homeostasis through the activity of the autonomic nervous system [18–27]. We therefore, suggest that the mesencephalic locomotor regions are closely linked to skeletal muscle activity through melanocortin-sympathetic signals.

In yet other studies, the neurotropic tracer microinjections are reported to be very widely used in brain mapping research [7, 15, 28–39]. The transneuronal tracer pseudorabies virus (PRV) was used to investigate whether connections from the gastrocnemius muscle to the mesencephalic locomotor region (MLR), which is composed of the pedunculopontine nucleus (PPTg) and the adjacent cuneiform nucleus (CnF) [40], are maintained by largely separate populations of neurons. The retrograde tracer PRV-614, was injected into the gastrocnemius muscle of MC4R-green fluorescent protein (GFP) transgenic mice after receiving a complete spinal cord transection below the L2 level using an electrocautery [16, 41, 42]. The aim of the present study was therefore, to use PRV-614 in order to trace the melanocortin-sympathetic neural pathways from pedunculopontine tegmental nucleus and cuneiform nucleus to the gastrocnemius muscle in spinally transected transgenic mice.

## RESULTS

### Specific expression of PRV-614 in the pedunculopontine nucleus areas

**Figure 1:**
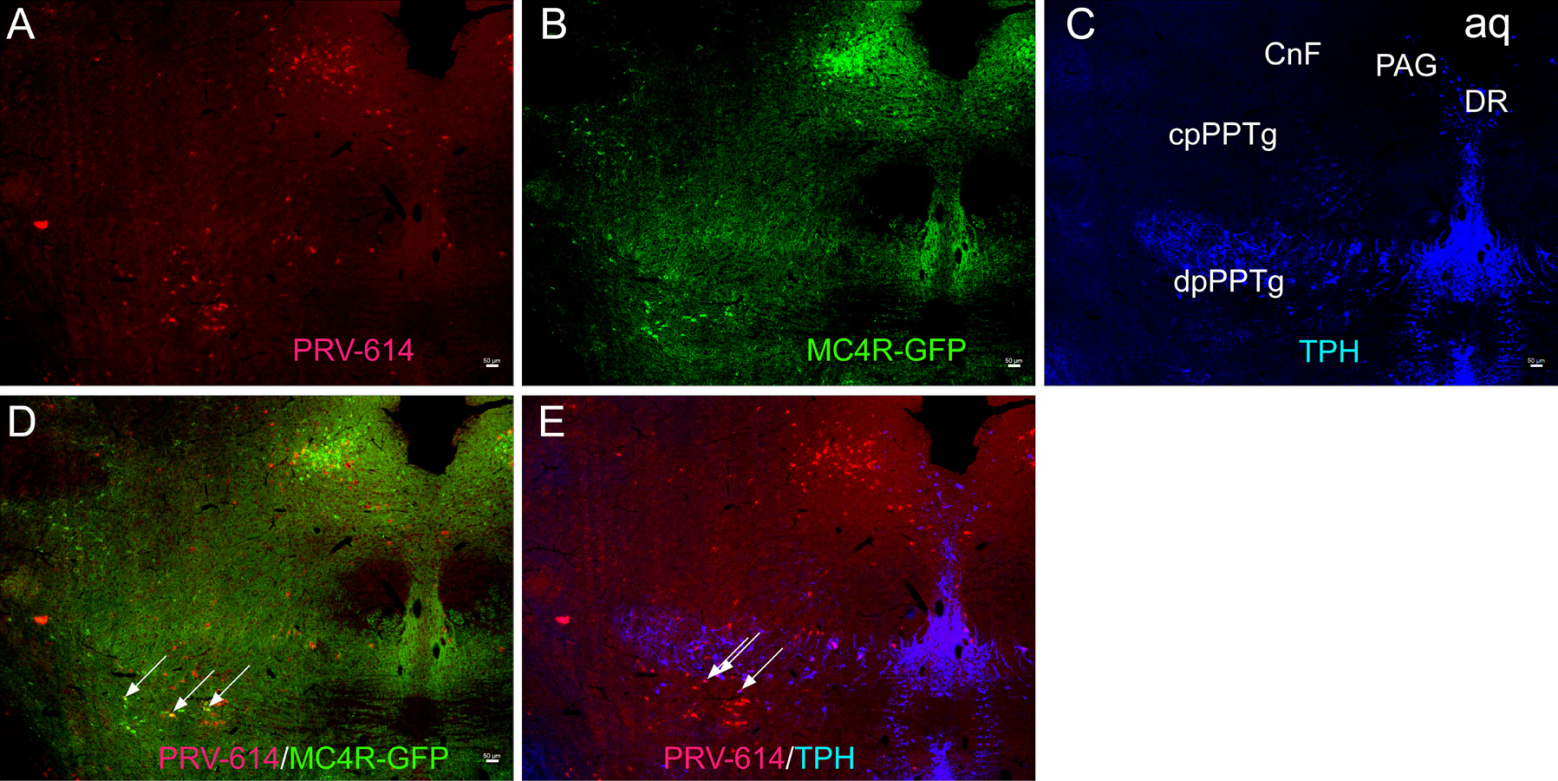
Micrographs illustrating sections through the CnF and caudal levels of the edunculopontine tegmental nucleus (PPTg) (**A**) Sections immunoreacted for PRV-614 (red). (**B**) Sections immunoreacted for MC4R-GFP (green). (**C**) Sections immunoreacted for TPH (blue). (**D**) Sections immunoreacted for PRV-614/MC4R-GFP dual labeled neurons (yellow, some are indicated by white arrows). (**E**) Sections immunoreacted for PRV-614/TPH dual labeled neurons (pink, some are indicated by white arrows). After a complete spinal cord transection, injections of PRV-614 into the gastrocnemius muscle resulted in retrograde infection of neurons in the CnF, cpPPTg and dpPPTg, and PRV-614/MC4R-GFP and PRV-614/TPH dual labeled neurons were detected in the dpPPTg. In contrast to the dpPPTg, we didn't detect dual labeled neurons in the CnF and cpPPTg. PRV-614, pseudorabies virus-614; CnF, cuneiform nucleus; Aq, aqueduct; DR, dorsal raphe; dpPPTg, the dissipated parts of PPTg; cpPPTg, the compact parts of PPTg; TPH, tryptophan hydroxylase. Scale bar: 50 μm.

Animals received a successful transection in the L2 spinal cord, as indicated by the lack of neurons immunopositive for PRV-614 in the ventral horn (VH) of the spinal cord. At 5 d after PRV-614 injection into the gastrocnemius muscle, retrograde infection of neurons were noticed in the spinal cord, medulla oblongata, and pedunculopontine nucleus areas. PRV-614-labeled neurons were found in the IML, RVLM, RVMM, and the dpPPTg but not in the CnF and cpPPTg (Figure 1A).

### MC4R and PRV-614 co-expression in the pedunculopontine nucleus areas

**Figure 2:**
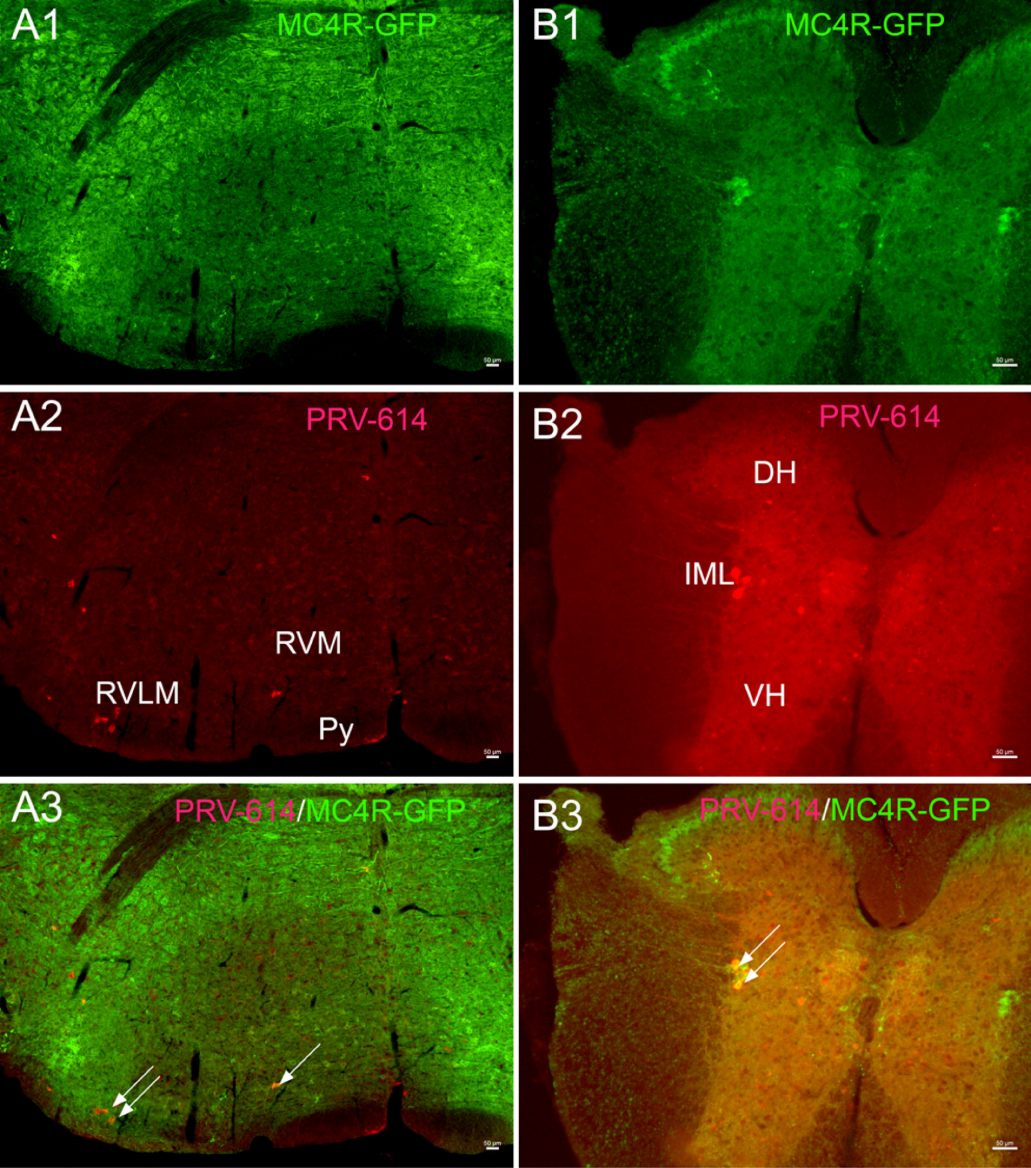
Gastrocnemius muscle cell groups target PRV-614 positive neurons (red) of the rostral ventral medulla (**A**) and spinal cord (**B**) levels. (**A1** and **B1**) Sections immunoreacted for MC4R-GFP (green). (**A2** and **B2**) Sections immunoreacted for PRV-614 (red). (**A3** and **B3**) Sections immunoreacted for PRV-614/MC4R-GFP dual labeled neurons (yellow, some are indicated by white arrows). After a complete spinal cord transection, injections of PRV-614 into the gastrocnemius muscle resulted in retrograde infection of neurons in the RVLM, RVMM, and IML. In contrast to the IML, we didn't detect labeled neurons in the VH in spinally transected transgenic mice. PRV-614, pseudorabies virus-614; IML, intermediolateral column; Py, pyramidal tract; RVLM, rostral ventrolateral medulla; RVMM, rostral ventromedial medulla; VH, ventral horn. Scale bar: 50 μm.

We examined GFP expression in the MC4R-GFP reporter mouse and observed a large number of GFP-positive neurons in the IML, medulla, and pedunculopontine nucleus areas. PRV-614/MC4R-GFP and PRV-614/TPH dual labeled neurons were found in the dpPPTg (Figure 1D, 1E and not in the CnF and cpPPTg (Figure 1E) after complete spinal cord transection. Dual-labeled PRV-614/MC4R-GFP neurons were coexpressed in the RVLM, RVMM, and IML (Figure 2, yellow, some are indicated by white arrows) but not in the VH (Figure 2). The percentage of PRV-614-colocalization in MC4R-GFP-positive neurons of the dpPPTg was 26.7%–30%, whereas that of PRV-614-colocalization in TPH-positive neurons of the dpPPTg was 20%–30%.

## DISCUSSION

In the present study, we utilized PRV-614 and MC4R-GFP mouse model to demonstrate that in spinally transected transgenic mice, the caudal pedunculopontine tegmental nucleus may be involved in the regulation of skeletal muscle activity of the melanocortin-sympathetic pathway. The principal findings of this study were: (1) specific midbrain regions, particularly the caudal pedunculopontine tegmental nucleus (Figure 1A), contained a substantial number of neurons that were infected 5 days following injections of PRV-614 into the gastrocnemius muscle. (2) PRV-614-labeled neurons were found in the IML, RVLM, RVMM, but not in the CnF and cpPPTg. (3) PRV-614/MC4R-GFP and PRV-614/TPH dual labeled neurons were detected in the dpPPTg after a complete spinal cord transection.

Considering that the parasympathetic nervous system failed to provide any innervations to limb muscles [16] and the spinal cord was transected rostral to the gastrocnemius motoneuron pool, we could speculate that the caudal pedunculopontine tegmental nucleus neurons were infected with PRV-614 via the sympathetic nervous system. Another explanation could be that there existed a direct neuronal circuit from the caudal pedunculopontine tegmental nucleus to skeletal muscle via the sympathetic pathway after the spinal cord transection. It is also evident from the graphs in Figure 1 that very few viral-infected cells were detect in the CnF (Figure 1A). Alternatively, it may reflect that the CnF does not play a major role in the regulation of skeletal muscle tone of the sympathetic pathway, which was in agreement with a previous report [43].

Interestingly, graphs in Figure 1A indicate that relatively small numbers of MC4R-GFP-labeled cells were scattered in the CnF whereas the caudal pedunculopontine tegmental nucleus showed moderate to high levels of MC4R-GFP immunoreactivity. Previous investigations have reported the coexpression of ChAT immunoreactivity and MC4R expression in the peduncular pontine tegmental nucleus [9]. Based on these data, we can speculate that ChAT immunoreactivity mainly exists in the caudal pedunculopontine tegmental nucleus rather than in the CnF. Other studies have found similar results in which the cholinergic neurons were preferentially distributed in the area corresponding to the inhibitory region (PPTg) rather than the locomotor region (CnF) [44].

A further feature of the results confirms that PRV-614/MC4R-GFP and PRV-614/TPH double-labeled neurons were observed in the dissipated parts of pedunculopontine tegmental nucleus (dpPPTg) (Figure 1D, 1E), but not in cpPPTg and CnF. On this basis, we could further speculate that CnF and cpPPTg neurons do not regulate skeletal muscle activity via the melanocortin-sympathetic pathway unlike dpPPTg neurons. In essence, these data indicate that projections from the caudal pedunculopontine tegmental nucleus (dpPPTg and cpPPTg) and cuneiform nucleus to skeletal muscle arise from largely separate populations of neurons. Therefore, other interpretations should be considered, particular that deep brain stimulation of the caudal pedunculopontine tegmental nucleus could be involved in melanocortin-sympathetic, serotonergic and motor pathways.

Moreover, we found that PRV-614/MC4R-GFP neurons were in several portions of the medulla oblongata including the rostral ventrolateral, ventromedial medulla (RVLM and RVMM) (Figure 2A), and the spinal cord (Figure 2B), which were consistent with previous reports [45] This suggests that there are direct connections between RVLM premotor neurons and sympathetic preganglionic neurons located in the IML.

**Figure 3:**
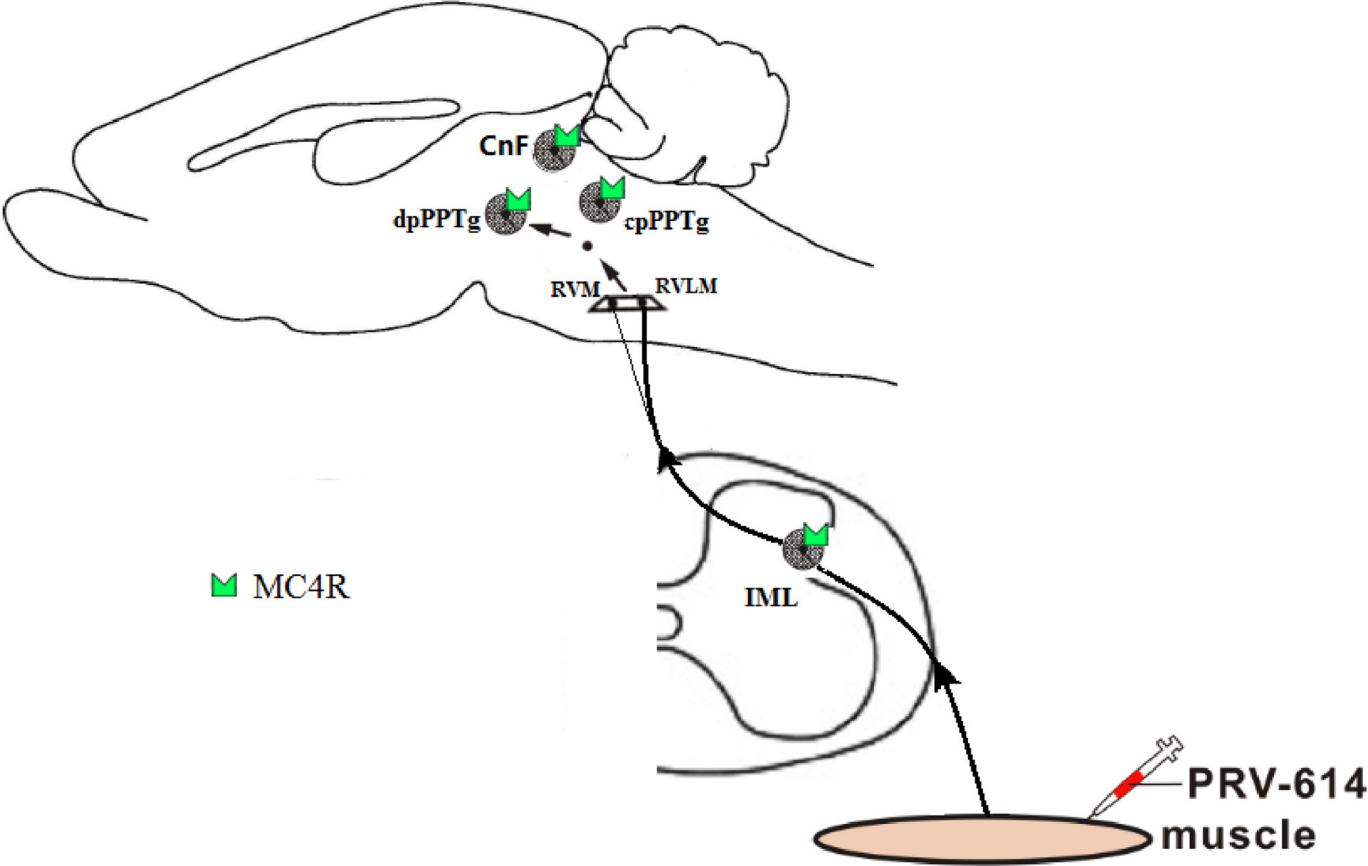
Summary diagram showed that the melanocortin-sympathetic pathway between the dpPPTg and skeletal muscle Neurons within the dpPPTg send projections to the rostral ventral medulla (e.g., RVLM premotor neurons), which in turn projects to the sympathetic preganglionic neurons within the IML, which control skeletal muscle activity regulated by melanocortin- sympathetic signals. PRV-614, pseudorabies virus-614; dpPPTg, the dissipated parts of PPTg; IML, intermediolateral column; RVLM, rostral ventrolateral medulla; RVMM, rostral ventromedial medulla. Some drawings were taken from HB Xiang (Brain 2013).

In conclusion, based on previous studies and our results, we can speculate that a hierarchical pathway has neurons within the caudal pedunculopontine tegmental nucleus that send projections to the RVLM, which in turn project to sympathetic preganglionic neurons within the IML, that regulate muscle blood flow through melanocortin-sympathetic signals (Figures 2 and 3). In addition, MC4Rs expressed in CnF and cpPPTg neurons do not regulate skeletal muscle tone via melanocortin-sympathetic pathways unlike MC4Rs in dpPPTg. Further research will be necessary to test the validity of our findings.

## MATERIALS AND METHODS

### Animals care

Experiments were carried out on transgenic MC4R-GFP mice, which were genotyped as described by Rossi and colleagues [20]. Mice were kept under controlled conditions (24 ± 0.5°C, 12 h alternating light-dark cycle, food and water ad libitum). Experimental procedures and protocols used in this study were approved in advance by the Animal Care and Use Committee of Tongji hospital, Huazhong University of Science & Technology (No. TJ-A20150805). This study also adhered to the guidelines of the U.K. Animals (Scientific Procedures) Act, 1986 and associated guidelines, the European Communities Council Directive of 24 November 1986 (86/609/EEC).

### Spinal transection

Male transgenic MC4R-GFP mice, weighing 25–30 g, were used in the experiments. The L2 spinal transection was performed in a manner similar to that described in previous reports [16]. Mice were provided analgesia based on a mixture of ketamine and ketoprofen. Following spinal transection, animals had their lower body paralyzed, and were maintained in a special cage so as to have access to food and water [16]. Gentle massage of the abdomen was operated to aid urination every 6 h [16].

### Microinjection of virus into the gastrocnemius muscle

PRV-614 was microinjected into the gastrocnemius muscle at day 3 after spinal cord transection on male transgenic MC4R-GFP mice in a manner similar to previous reports [16]. In brief, 2 μl injections of PRV-614 were inserted into the gastrocnemius muscle (0.5 μl per injection at 4 injection sites per one mouse). After every injection, the needle was kept *in situ* for 2 minutes to limit the spread of PRV-614.

### Perfusion and fluorescence immunohistochemistry

After a survival time of 5 days, mice were deeply anesthetized, and the spinal cord and brain were removed and post-fixed in 4% paraformaldehyde-borate. Tissue sections were processed for GFP (1:1,000; Molecular Probes, Eugene, OR) fluorescence immunohistochemistry according to standard protocols [29, 46]. For TPH IHC, the first antibody was sheep anti-TPH (1:2000; Chemicon International, Temecula, CA) and the second antibody included biotinylated donkey anti-sheep IgG (H + L) (Lot no.68003, Jackson ImmunoResearch Lab., West Grove, PA) and streptavidin Alexa Fluor 350 conjugate (Lot no.49248A, S-11249, Invitrogen, Molecular Probes, Eugene, OR).

### Tissue analysis

Immunofluorescence photomicroscope was visualized by using an Olympus IX81 photomicroscope. PRV-614-positive neurons were identified with red fluorescence, MC4R-GFP- expressing neurons were recognized by green fluorescence, and TPH-expressing neurons were recognized by blue fluorescence. The regions in which positive cells were located were defined with reference to the atlases of Franklin KB and Paxinos G [47].
